# Two-Stage Lumbar Dynamic Stabilization Surgery: A Comprehensive Analysis of Screw Loosening Rates and Functional Outcomes Compared to Single-Stage Approach in Osteopenic and Osteoporotic Patients

**DOI:** 10.3390/diagnostics14141505

**Published:** 2024-07-12

**Authors:** Mehdi Hekimoglu, Mehmet Yigit Akgun, Hidir Ozer, Ahmet Tulgar Basak, Ege Anil Ucar, Tunc Oktenoglu, Ozkan Ates, Ali Fahir Ozer

**Affiliations:** 1 Department of Neurosurgery, American Hospital, Istanbul 34365, Turkey; mehdih@amerikanhastanesi.org (M.H.); basak_ahmet@hotmail.com (A.T.B.); tuncoktenoglu@gmail.com (T.O.); 2 Department of Neurosurgery, Koc University Hospital, Istanbul 34010, Turkey; myigitakgun@gmail.com (M.Y.A.); eucar17@ku.edu.tr (E.A.U.); oates@kuh.ku.edu.tr (O.A.); 3 Department of Neurosurgery, Ordu University, Ordu 52200, Turkey; hidirozer@hotmail.com; 4 Spine Center, Koc University Hospital, Istanbul 34010, Turkey

**Keywords:** clinical outcomes, dynamic spine stabilization, osteopenia, osteoporosis, screw loosening, two-stage surgery

## Abstract

Background: Dynamic lumbar stabilization aims to preserve spinal movement, offering stability and controlled motion. However, screw loosening, especially in patients with osteopenia and osteoporosis, remains challenging. Method: Between 2018 and 2022, a retrospective analysis was conducted on a total of 119 patients diagnosed with osteopenia and osteoporosis who underwent spinal dynamic instrumentation surgery. These patients were categorized into two groups: single-stage surgery (*n* = 67) and two-stage surgery (*n* = 52). Over the 48-month follow-up period, the occurrence and percentage of screw loosening were examined at each surgical level per patient, as well as by screw location (pedicular, corpus, tip). Clinical outcomes were evaluated using Visual Analog Scale (VAS) and Oswestry Disability Index (ODI) scores. Results: Total screw loosening rates were significantly lower in the two-stage group (2.83%) compared to the single-stage group (14.63%, *p* < 0.001). Patient-based loosening occurred in 5 patients (9.6%) in the two-stage group and 16 patients (23.9%) in the single-stage group. Loosening rates were lower in the two-stage group at L2 (7.78%, *p* = 0.040), L3 (5.56%, *p* < 0.001), L4 (8.89%, *p* = 0.002), and L5 (10.00%, *p* = 0.006), but higher at S1 (21.11%, *p* = 0.964), T12 (15.56%, *p* = 0.031), and iliac levels (15.56%, *p* = 0.001). Pedicular section exhibited the highest loosening (37 cases). VAS and ODI scores improved significantly in both groups, with better outcomes in the two-stage group at the 48. months (*p* < 0.001). Conclusions: The two-stage surgical approach significantly reduces screw loosening in patients with osteopenia and osteoporosis undergoing dynamic stabilization surgery, offering enhanced stability and better clinical outcomes.

## 1. Introduction

Dynamic stabilization is a technique employed in spinal surgery that aims to preserve spinal functionality while addressing conditions such as instability and degenerative disc disease [1]. Unlike traditional fusion methods that restrict spinal motion, dynamic stabilization offers stability and controlled movement, thereby minimizing the risk of complications associated with rigid instrumentation [2,3]. Specifically, the Dynesys system, one of the common dynamic stabilization systems, utilizes pedicle screws connected by flexible rods to provide stability while allowing controlled motion. This system aims to mimic the biomechanical properties of the intact spine, reducing risks like adjacent segment degeneration and pseudoarthrosis [4]. However, screw loosening remains a significant challenge in dynamic stabilization procedures, potentially compromising the stability of the spinal construct and hindering long-term outcomes [5]. Factors such as bone quality, screw design, and surgical technique are crucial in mitigating this risk [6]. The literature indicates that higher extraction torque is frequently observed in the pedicular portion of screws during spinal stabilization procedures, attributable to the denser bone quality in this region, which is subject to greater mechanical stress and load bearing [7]. Additionally, improper screw positioning or angulation during surgery can exacerbate the elevation of extraction torque [8,9,10,11].

Screw loosening is a particularly relevant concern for patients with osteoporosis. These individuals face a heightened risk due to compromised bone quality and reduced bone strength [12]. Addressing this issue is pivotal for ensuring the long-term success of dynamic stabilization procedures [13].

In response to this challenge, we previously proposed the two-stage surgical technique as a potential solution to reduce screw loosening rates and enhance patient outcomes [14]. This study evaluates the efficacy of the two-stage dynamic stabilization approach in mitigating screw loosening rates. Through a comparison of clinical and radiological outcomes, screw loosening rates, and complication profiles between osteopenic/osteoporotic patients undergoing single-stage and two-stage dynamic stabilization surgery, we aim to assess the transformative potential of the two-stage surgical approach. Additionally, we analyzed the portion where screw loosening occurs, specifically within the pedicular section, corpus section, and screw tip.

The primary objective of dividing patients into two groups was to compare the clinical and radiological outcomes of single-stage versus two-stage dynamic stabilization approaches. This approach allowed for a comprehensive evaluation of the efficacy and safety of each surgical technique across a diverse patient population. The selection criteria for group assignment included the severity of osteoporosis, the extent of spinal instability, and the surgeon’s assessment of the patient’s overall health and ability to withstand surgery. The effort was concerted to ensure that T-scores, group numbers, gender distribution, and surgical levels were balanced as much as possible, aiming to avoid any bias that could affect the results.

## 2. Methods

### 2.1. Study Approval

In this study, all procedures performed were in accordance with the ethical standards of the institutional and national research committee and with the 1964 Helsinki Declaration and its later amendments or comparable ethical standards. Informed consent was obtained from all participants included in the study. Ordu University Faculty of Medicine (Ordu-Turkey) Clinical Research Ethics Committee approved this study.

### 2.2. Study Design

We conducted a retrospective analysis involving 119 patients who underwent spinal instrumentation surgery with Dynesys dynamic systems between 2018 and 2022 during a 48-month evaluation. Inclusion criteria for all patients were a previous diagnosis of osteopenia and/or osteoporosis and a T-score lower than −1.5 at the time of surgical decision. The surgical indications for the patients included in this cohort were spinal lumbar stenosis, degenerative spondylolisthesis, degenerative deformity, painful degenerative disc disease, painful degenerative spondylolysis, and spinal instability due to previous discectomy surgeries. The history of steroid use was also considered. The demographic information, medical history, surgical details, postoperative outcomes, radiological data, and complications were recorded.

Study Groups: Patients were divided into two groups based on the surgical approach. The first group underwent single-stage dynamic stabilization surgery, while the second group underwent a two-stage approach with rod placement performed six months after the initial operation.

Criteria for Group Assignment: The primary objective of dividing patients into two groups was to compare the clinical and radiological outcomes of single-stage versus two-stage dynamic stabilization approaches without predetermined criteria for group assignment. This approach allowed for a comprehensive evaluation of the efficacy and safety of each surgical technique across a diverse patient population. As much as possible, group numbers, gender distribution, and surgical levels were balanced.

### 2.3. Radiological Analysis

Bone density T scores were determined using dual-energy X-ray absorptiometry before surgery through bone scintigraphy. All patients underwent MRI and CT scans of the pathological region and standing anteroposterior and lateral X-rays. During the follow-ups, patients were monitored at 6th, 12th, 24th, and 48th months and annually thereafter. CT scans were used to assess screw loosening and osteointegration, with particular attention to signs such as radiopacities or radiolucency around screw heads, changes in screw position, and displacement of adjacent bone fragments or hardware components. Moreover, the distribution of screw loosening was analyzed by categorizing it into three parts based on the location of the screw on CT scans: the pedicular section, the corpus section, and the screw tip. The spinal level of screw loosening was recorded for each patient, enabling a comparison of screw loosening rates between the two surgical techniques. Additionally, the functional outcomes of patients in both groups were evaluated using the Visual Analog Scale (VAS) and Oswestry Disability Index (ODI), with scores recorded at each follow-up visit.

### 2.4. Surgical Technique and Nuances

Dynamic stabilization was performed in all cases. Stabilization was performed in the prone position using the Wiltse method. The operations were performed under spinal or general anesthesia according to the patient’s factors and the surgeon’s preference. In the two-stage surgical approach, spinal anesthesia was preferred more because the time was divided into two stages, and it was observed that the comfort level of the patients increased. If the patient did not require decompression, a transpedicular screw system was inserted percutaneously under fluoroscopic guidance, especially in the first stage of two-stage surgeries. If the patient needed decompression, the paravertebral muscles were dissected in the areas to be decompressed, and the screws were placed with the Wiltse method after the decompression process was completed. If perioperative instability was observed after decompression, temporary rods were placed only in the decompressed segment and side. Thus, it was aimed to risk one or two screws in case of a possible loosening. In the two-stage operations, patients were discharged after screw placement. Patients were evaluated with CT after an average of 6 months. In addition, osteoporosis/osteopenia treatment was initiated with endocrinology consultation in all patients during this period. After it was determined that the osteointegration of screws was complete, if necessary, primary pathologies were fixed, and then the Dynesys system’s rods were placed and connected. It is necessary to restore impaired lumbar lordosis in patients. To bring the forward slipped sagittal vertical axis back to the sacrum, patients with flat back deformity were placed in a lordotic position on the table and controlled with a C-arm. If necessary, iliac long segment dynamic system stabilization was performed to ensure the continuity of the lordotic position.

### 2.5. Statistical Analysis

NCSS (Number Cruncher Statistical System) 2020 Statistical Software (LLC, Kaysville, UT, USA) program and GraphPad (Prism) version 9 (GraphPad Software, San Diego, CA, USA) were used for the statistical analyses. Ordinary one-way ANOVA analysis was used for the comparison of VAS and ODI scores between groups. We used an independent samples *t*-test to compare age and T scores between the two groups. We used Pearson’s chi-square test, Fisher’s exact test, and Fisher–Freeman–Halton test for the evaluation of categorical variables. The results were presented as mean ± standard deviation or frequency (%). Statistical significance was set at *p* < 0.05.

## 3. Results

1. Patient Characteristics

The study examined 119 cases, comprising 81 (68%) females and 38 (31.9%) males, with ages ranging from 42 to 81 years and a mean age of 58.08 years. The follow-up duration was 48 months. All participants had a pre-existing diagnosis of osteoporosis, with a mean T-score of −2.39 ± 0.47 in the two-stage procedure group and −2.45 ± 0.45 in the single-stage procedure group at presentation. Among the participants, 67 patients underwent dynamic stabilization by a single-stage approach, while 52 (43.70%) patients underwent dynamic stabilization by a two-stage approach (Table 1).

2. Distribution of Screw Loosening across Surgical Levels and Sections

The distribution of patients across surgical levels and the occurrence of screw loosening were assessed for each surgical level and procedure group. In the single-stage group, 16 (23.9%) patients required revision surgery for screw loosening, whereas revision surgery was required in only 5 (9.6%) patients in the two-stage group (Table 2). The most prevalent occurrences of screw loosening were observed at the S1 level (19 events) and the iliac level (14 events), followed by the T12 level (14 events) and the L1 level (10 events). There was no occurrence of screw loosening at the T9 and T10 levels. Furthermore, significantly lower occurrences of screw loosening were observed at the L2, L3, L4, and L5 levels (*p* = 0.040, *p* < 0.001, *p* = 0.002, *p* = 0.006, respectively) (Table 3).

Analyzing the distribution of screw loosening by screw section, it was observed that the pedicular section accounted for the highest incidence of screw loosening, comprising 37 events (41.57%), followed closely by the corpus section, with 33 events (37.07%). The tip section had the lowest incidence of screw loosening, totaling 20 events (21.35%). There was no significant difference between screw loosening of pedicular, corpus regions, both in total and at each level. Detailed distribution of screw loosening by screw section and surgical level is provided in Table 3.

3. Rate of Screw Loosening

A total of 929 screws were assessed among 119 patients, revealing 90 instances of screw loosening, resulting in an overall screw loosening rate of 9.69%. Within the single-stage group, 79 screws out of 540 (14.63%) experienced loosening, while the two-stage group exhibited a significantly lower rate of screw loosening, with only 11 screws out of 389 (2.83%) (*p* < 0.0001, RR: 0.88, 95% CI: 0.84–0.91) (Table 4). Screw loosening requiring revision per patient within 48 months was significantly higher in the single-stage group (23.9%) compared to the two-stage group (9.6%), with a *p*-value of 0.026. Additionally, the number of loosened screws was significantly greater in the single-stage group (14.63%) than in the two-stage group (2.83%), with a *p*-value of 0.001 (Table 1).

4. Clinical Outcomes and Complications

In terms of clinical outcomes, both single-stage dynamic stabilization and two-stage dynamic stabilization groups exhibited significant improvement in Visual Analog Scale (VAS) and Oswestry Disability Index (ODI) scores (*p* < 0.001). However, the scores in the two-stage surgery group were slightly higher at 6 months. However, from the 6-month mark and onwards, the VAS and ODI scores in the two-stage surgery group consistently improved and surpassed those in the single-stage surgery group (Table 5).

Regarding perioperative findings, the mean operating time was 211.2 ± 37.3 min for the single-stage group and 191.7 ± 41.4 min for the two-stage group at the first stage. The mean intraoperative blood loss was 591.25 ± 246.3 mL for the single-stage group and 357.33 ± 121.6 mL for the two-stage group (Table 1).

**Figure 1:**
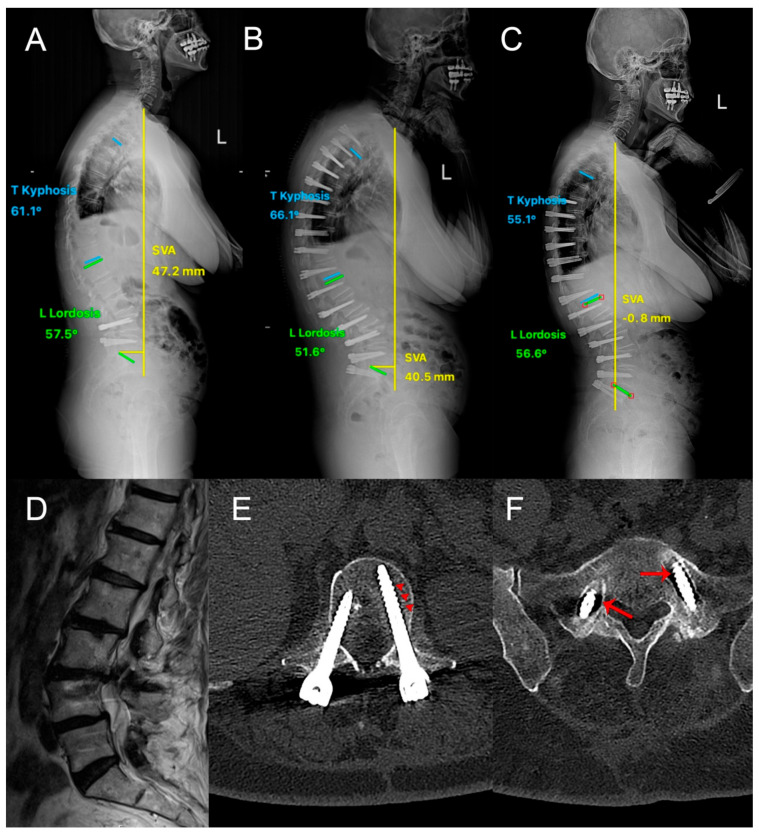
A 69-year-old female patient with osteoporosis (T: −2.8) who has been previously operated for lumbar spinal stenosis at L4–L5 presented with neurological claudication and sensory loss in both extremities. (**A**) Preoperative lateral standing X-ray showing sagittal imbalance. (**D**) Preoperative sagittal T2W MRI showing adjacent segment degeneration and spinal stenosis at L3–L4 and L2–L3. Two-stage dynamic stabilization with the Dynesys system was performed. Standing lateral X-ray (**B**) after 1st operation and (**C**) after 2nd operation, showing significant improvement in sagittal alignment. (**E**) Postoperative (2nd operation) axial CT scan showing successful osteointegration at L1 level, and (**F**) postoperative (2nd operation) axial CT scan showing marked screw loosening at S1 level. The areas of radiolucencies, as indicated by arrows, demonstrate screw loosening, while arrowheads highlight osteointegration.

**Figure 2:**
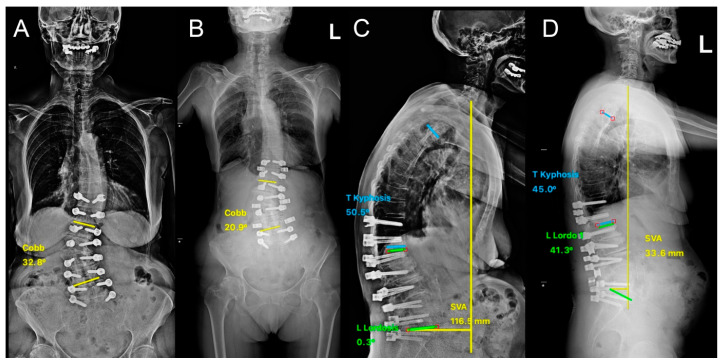
Patient with spinal stenosis and mixed coronal-sagittal deformity presented to the clinic with lower back pain and neurogenic claudication. The patient was previously diagnosed with osteoporosis (T: −2.9). Two-stage dynamic stabilization with Safinaz and PEEK rods was performed. Standing anteroposterior X-rays (**A**) before and (**B**) after the second-stage surgery showing improvement in the coronal deformity. Standing lateral X-rays (**C**) before and (**D**) after the second-stage surgery demonstrate significant improvement in the sagittal alignment.

**Figure 3:**
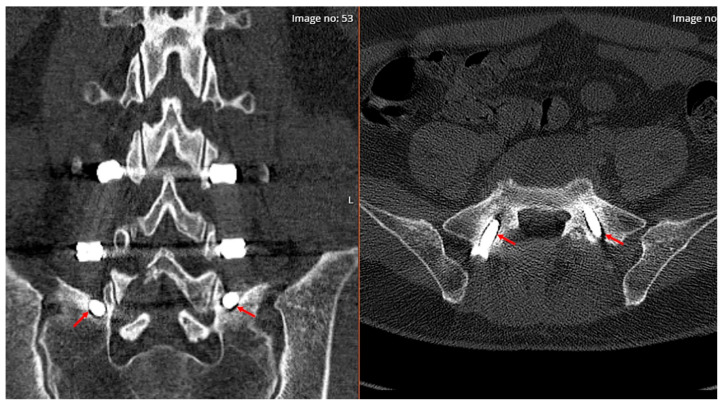
56-year-old female patient with osteoporosis (T-score: −2.6) who has previously undergone surgery for lumbar spinal degenerative disease using a single-stage surgical technique. At the 3-month follow-up lumbar tomography due to back pain, early-stage loosening of the S1 screws on both sides is observed. (Red arrows). These findings indicate early screw loosening, particularly due to increased load on the screws in osteoporotic patients.

**Figure 4:**
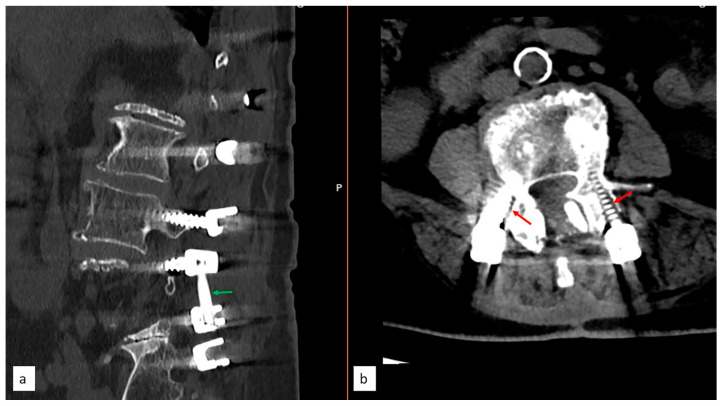
(**a**). 78-year-old female patient with osteoporotic T-score underwent surgery using a two-stage technique. During the first-stage surgery, decompression at the L4–L5 level was conducted. To prevent the potential development of segmental instability, a temporary peek rod was placed at the level of L4–L5 (Green arrow). (**b**). After 6 months of osteoporosis treatment, when she presented for the second-stage surgery, a CT scan revealed acceptable osteointegration, but bilateral L4 screws appeared loosened. (Red arrows) These findings supported the theory that surgeries performed in a single stage may cause overload on the screws and lead to loosening.

Additionally, in the single-stage group, one (1.5%) subcutaneous hematoma, one (1.5%) superficial tissue infection, and two (2.99%) instances of adjacent segment degeneration were observed, but none required revision surgery. In the two-stage group, one (1.8%) superficial tissue infection and one (1.8%) instance of adjacent segment degeneration without clinical findings were observed (Table 1). Selected cases from the cohort are illustrated in Figure 1, Figure 2, Figure 3 and Figure 4.

## 4. Discussion

This study aimed to evaluate the efficacy of two-stage dynamic stabilization surgery compared to the single-stage approach in reducing screw loosening rates and improving clinical outcomes in patients with osteopenia and osteoporosis. Our findings indicate that the two-stage surgical approach significantly reduces the incidence of screw loosening and enhances patient outcomes, as measured by VAS and ODI scores.

The reduction in screw loosening rates observed with the two-stage approach has significant clinical implications. Screw loosening can lead to instability, pain, and the need for revision surgery, which can be particularly challenging in osteoporotic patients [15]. By minimizing this complication, the two-stage approach not only enhances stability but also potentially reduces the overall healthcare burden associated with revision surgeries and prolonged recovery times.

Furthermore, the improved clinical outcomes in terms of VAS and ODI scores indicate better pain management and functional recovery in patients undergoing the two-stage surgery. This finding is crucial for improving the quality of life in osteoporotic patients, who are often more vulnerable to postoperative complications and slower recovery [16].

Previous studies have highlighted the challenge of screw loosening in dynamic stabilization procedures, particularly in patients with compromised bone quality, such as those with osteopenia and osteoporosis [17,18]. Our study aligns with these findings. For instance, Wu JC (2011) and Kou et al. (2015) reported screw loosening rates of 4.7% per screw and 19.8% per patient, and 8.2% per screw and 20.4% per patient, respectively, in single-stage dynamic stabilization surgeries [19,20]. These rates are similar to our observed rates of 14.63% per screw and 23.9% per patient. In contrast, our study demonstrates a notable reduction in screw loosening rates with the two-stage approach, showing a rate of 2.83% per screw and 9.6% per patient. This significant improvement underscores the potential of the two-stage approach.

Back pain symptoms often result from abnormal load distribution and movement of the spine. Many patients report postural or positional pain as the main symptom. Consequently, there is a growing notion of mimicking physiological conditions in load transmission during surgical procedures to achieve the most optimal outcomes. In cases presenting with radicular symptoms and neurogenic claudication, decompression surgery may become necessary due to spinal canal narrowing and root compression. It is important to note that decompression can impact spinal stability, sometimes requiring additional stabilization [21,22].

Limiting spinal motion through fusion surgery may lead to complications such as adjacent segment degeneration, infection, and pseudoarthrosis. This is particularly relevant in elderly patients, where adjacent segment degeneration or intervertebral disc disorders may manifest without providing significant clinical benefits from lumbar fusion [23]. Dynamic stabilization emerges as an optimal treatment for patients experiencing slow-developing instability without fixed spinal alignment impairments. This approach offers various advantages, including early mobilization, reduced pain, quicker reintegration into social activities, and minimizing potential complications, such as adjacent segment degeneration commonly associated with the use of rigid instrumentation [21,22,23].

The primary challenge associated with dynamic stabilization systems is screw loosening. Various factors contribute to this issue, encompassing biomechanical forces, stress concentration, bone quality, surgical technique, and implant design [24,25]. Numerous studies have proposed strategies to mitigate screw loosening rates, but it remains a persistent issue, particularly in elderly and osteoporotic cases. The neutral zone within the motion segment gradually expands, leading to increased instability over time, resulting in back pain for patients [14,15,16,17,18,19,20,21,22,23,24,25,26,27,28].

The two-stage surgical approach for posterior spine stabilization addresses critical factors contributing to the failure of traditional single-stage surgeries. Primary stabilization provided by initial screws and rods is crucial, but in chronic instability, secondary stability, i.e., integration of screws into bone, is essential for long-term success. Failure often arises due to poor bone quality and excessive mobility at the unstable segment, leading to screw loosening and instrument failure [29]. In conditions like osteoporosis, rheumatoid arthritis, or ankylosing spondylitis, poor bone quality necessitates longer instrument constructs to distribute load per screw and prevent loosening. To mimic bone healing according to Wolff’s Law, some load must be placed on screws without compromising primary stability; inadequate primary stability may lead to pseudoarthrosis and instrument failure [30]. In a two-stage approach, initial screw placement reduces the load on screws, allowing for easier acceptance by bone. Remodeling between days 21–35 strengthens secondary stability over months, enabling solid fusion [31]. Lumbar screws share load with the spine, allowing immediate post-surgery mobility [32]. The second stage, performed after 4–6 months, involves attaching rods to solidly fused screws, ensuring stable long-term stabilization. This approach optimizes both primary and secondary stability, which is crucial for successful spine stabilization in osteoporotic patients.

In our prior work, we developed a two-stage surgical technique tailored for patients requiring spinal stabilization, especially those with a heightened risk of instrument failure [14]. In this study, we further evaluated the screw loosening rates of two-stage dynamic stabilization over a longer period of 48 months compared to the traditional single-stage stabilization approach. Only 5 (9.6%) patients required revision surgery due to screw loosening in the two-stage surgery group, whereas 16 (23.9%) patients required revision surgery in the single-stage group for the same reason. The explanation for screw loosening in the two-stage surgical approach can be as follows: in the patient group that underwent two-stage surgery, decompression was necessary in specific segments for certain patients. To address potential issues stemming from segmental instability until the second stage of surgery, a temporary rod was inserted into the segment where decompression was performed. Screw-loosening cases were also observed precisely in the areas where the temporary rod was placed. This supports the theory that “placing a rod in a single stage, particularly in osteoporotic spines, may heighten the risk of screw loosening due to increased load on the screws during single-stage surgery”. These findings underscore the critical importance of allowing implants ample time to seamlessly integrate into the surrounding bone, thereby facilitating gradual loading of the implant screws and enhancing overall stability. Consequently, this contributes to improved osteointegration rates in a two-stage approach when using a dynamic stabilization system.

The susceptibility of screws is mostly attributed to the distribution of biomechanical forces applied to the pedicular area of the spine [33,34]. This region experiences significant stresses during spinal movements, and recurrent loading forces can induce micro-motion at the screw–bone interface, potentially causing loosening over time. Furthermore, the pedicle has been identified as the primary load-bearing structure of the vertebra, responsible for transmitting applied loads to the spine’s vertebral body [35,36]. Although the pedicle region was the most prone to loosening, we did not find any significant difference in screw loosening when comparing the loosening rates of the pedicle and corpus regions.

The current study also revealed a substantial variation in screw loosening rates across different surgical levels of the spine. The highest percentage of screw loosening was observed at the sacral S1 level, followed by the iliac, T12, and L1 levels. The sacrum, being a weight-bearing structure, undergoes significant load transfer, making biomechanical forces and stresses at the sacral level impact screws more profoundly than at other levels, resulting in a higher incidence of screw loosening [20,26,27]. Conversely, lower screw loosening rates were observed at other levels, such as L2, L3, L4, and L5, which may be attributed to differences in load-sharing and biomechanical factors specific to those segments under dynamic stabilization [37,38,39,40].

The significantly lower rates of screw loosening and favorable clinical outcomes in patients undergoing two-stage surgery highlight the enhanced stability, attributed in part to improved bone integration and osseointegration facilitated by the staged approach [41,42]. These findings underscore the critical role of osteointegration in enhancing stability and long-term outcomes in neurosurgical interventions [43]. Furthermore, one of the most notable advantages of two-stage surgery is its ability to reduce the need for future revision surgeries (Table 2)

The difference between our findings and the literature might stem from the fact that most studies evaluating screw loosening use rigid stabilization systems, whereas dynamic stabilization was used in this study. Therefore, the forces applied to each vertebral level and their distribution might be more homogenous in dynamic stabilization.

We believe that studies on stabilization techniques other than dynamic stabilization systems, such as surgical techniques using PEEK rods or rigid rods, should also be conducted to increase the reliability of our results. Nevertheless, since known forces are assumed to be applied to the screws in all stabilization techniques, it appears that the two-stage method could be beneficial for all types of stabilization methods. The two-stage approach allows for improved screw osteointegration before final stabilization, potentially leading to enhanced screw fixation and reduced micromovement that could cause loosening. This stepwise method may also contribute to the observed improvements in clinical outcomes [41,42].

This study provides a comprehensive analysis of screw loosening rates at each spinal level for both single-stage and two-stage surgical techniques, supported by clinical and radiological outcomes over a follow-up period of 40 months. The inclusion of a diverse patient population, including both osteopenic and osteoporotic patients, enhances the generalizability of the findings across different bone densities.

## 5. Limitations

Limitations of this study include its retrospective nature, which inherently introduces potential biases and limitations in the inclusion of data. The sample size could impact statistical power, suggesting the need for further investigations with larger cohorts. Additionally, while the study evaluates screw loosening rates and clinical outcomes, it does not comprehensively address other relevant factors influencing surgical success, such as patient comorbidities and surgeon experience. The follow-up duration may not capture long-term complications or outcomes beyond the study period. Future studies incorporating prospective designs, larger sample sizes, and longer follow-up durations are needed to validate the findings and address these limitations effectively. Investigating the biomechanical mechanisms underlying the improved outcomes with the two-stage approach, as well as the cost–effectiveness of the two-stage procedure compared to the single-stage approach, would also provide valuable insights for clinical decision-making.

## 6. Conclusions

This study highlights the efficacy of a two-stage surgical approach in mitigating screw loosening rates in osteopenic and osteoporotic patients undergoing dynamic lumbar stabilization with Dynesys systems. The findings demonstrate significantly lower instances of screw loosening with the two-stage technique compared to traditional single-stage surgery, indicating improved stability while producing favorable clinical outcomes. Given these findings, the two-stage surgical technique can be considered as an alternative treatment option for patients that are at high risk of screw loosening, particularly those with osteoporosis.

## Figures and Tables

**Table 1:** Screw loosening requiring revision per patient within 48 months was significantly higher in the single-stage group (23.9%) compared to the two-stage group (9.6%), with a *p*-value of 0.026. Additionally, the number of loosened screws was significantly greater in the single-stage group (14.63%) than in the two-stage group (2.83%), with a *p*-value of 0.001.

Characteristics	Two-Stage Procedure (*n* = 52)	Single-Stage Procedure (*n* = 67)	*p*-Value
Age (years)	57.09 ± 4.54	58.92 ± 4.84	^t^ 0.343
Age Groups			
- <50	2 (3.8%)	3 (4.5%),	^b^ 0.975
- 50–60	21 (40.4%),	26 (38.8%)	
- 60–80	29 (55.8%)	38 (56.7%)	
Gender			
- Female	34 (65.4%)	47 (70.1%)	^b^ 0.580
- Male	18 (34.6%)	20 (29.9%)	
Comorbidities			
- Steroid Use	9 (17.3%)	8 (11.9%)	^b^ 0.407
- Degenerative Disk Disease	43 (82.7%)	59 (88.1%)	
**T-score Mean**	−2.39 ± 0.47	−2.45 ± 0.45	^t^ 0.453
Age Groups (Steroid Users)			
<50 years	2 (22.2%)	3 (37.5%)	^f^ 0.142
50–60 years	3 (33.3%)	5 (62.5%)	
60–85 years	4 (44.4%)	0	
Age Groups (Degenerative Disk Disease)			
<50 years	0	0	
50–60 years	14 (32.6%)	21 (35.6%)	^b^ 0.750
60–85 years	29 (32.6%)	38 (64.4%)	
**T-score (Steroid Users)**			
<50 years	−2.10 ± 0.56	−2.18 ± 0.59	^ t ^ 0.564
50–60 years	−2.48 ± 0.20	−2.82 ± 0.23	^ t ^ 0.099
60–85 years	−2.23 ± 0.53	-	-
**T-score (Non-Steroid Users/Degenerative Disk Disease)**			
<50 years	-	-	-
50–60 years	−2.18 ± 0.41	−2.10 ± 0.22	^ t ^ 0.879
60–80 years	−2.52 ± 0.49	−2.62 ± 0.44	^ t ^ 0.534
Screw Length (mm)	45.8 ± 2.4	46.3 ± 2.1	^ t ^ 0.286
Screw Diameter (mm)	6.2 ± 0.3	6.1 ± 0.4	^ t ^ 0.654
Follow-up Period (months)	48	48	-
Complications			
- Superficial Tissue Infection	1 (1.8%)	1 (1.5%)	^ a ^ 1.000
- Screw loosening requiring revision surgery within 48 months follow-up per patient/percent	5 (9.6%)	16 (23.9%)	^ b ^ 0.048 *
- Number of screws loosened during 48 months follow-up - per screw/percent	11 (2.83%)	79 (14.63%)	^ b ^ 0.001 **
- Subcutaneous Hematoma	0	1 (1.5%)	^ a ^ 1.000
- Instances of Adjacent Segment Degeneration	1 (1.8%)	2 (3.0%)	^ a ^ 1.000
- Mean Operating Time/Min	191.7 ± 41.4 min	211.2 ± 37.3 min	
- Mean İntraoperative Blood Loss/Ml	357.33 ± 121.6 mL	591.25 ± 246.3 mL	

^a^ Fisher’s exact test, ^b^ chi-square test, ^f^ Fisher–Freeman–Halton test (data are represented as *n*(%)), ^t^ independent samples *t*-test (data are represented as mean ± standard deviation). * *p* < 0.05, ** *p* < 0.01.

**Table 2:** Distribution of screw loosening across surgical levels. The loosening status of screws between the two groups and the relationship between surgical levels have been evaluated. The distribution of loosening cases according to surgical levels and the number of loosening occurrences at each surgical level have been reported in detail.

Surgical Level	Number of Patients; *n*(%)	Two-Stage Procedure Patient Count by Surgical Level (*n* = 52)	Two-Stage Procedure Screw Loosening Patient Count No/Percent	Single-Stage Procedure Patient Count by Surgical Level (*n* = 67)	Single-Stage Procedure Screw Loosening Patient Count No/Percent
L1–S1	5 (4.2%)	1 (1.92%)	0	4 (5.97%)	3
L2–L4	5 (4.2%)	1 (1.92%)	0	4 (5.97%)	0
L2–L5	10 (8.4%)	3 (5.77%)	0	7 (10.45%)	0
L2–iliac	8 (6.72%)	4 (7.69%)	1	4 (5.97%)	2
L3–S1	21 (17.65%)	9 (17.31%)	0	12 (17.91%)	1
L3–iliac	5 (4.2%)	3 (5.77%)	1	2 (2.99%)	1
L4–S1	16 (13.45%)	7 (13.46%)	0	9 (13.43%)	0
T9–L5	7 (5.88%)	5 (9.62%)	0	2 (2.99%)	1
T10–iliac	13 (10.92%)	7 (13.46%)	1	6 (8.96%)	1
T6–S1	1 (0.84%)	1 (1.92%)	0	0 (0%)	0
T10–S1	13 (10.92%)	5 (9.62%)	0	8 (11.94%)	1
T11–S1	2 (1.68%)	1 (1.92%)	0	1 (1.49%)	1
L2–S1	9 (7.56%)	3 (5.77%)	0	6 (8.96%)	1
T11–iliac	4 (3.36%)	2 (3.85%)	2	2 (2.99%)	4
Toplam	119 patients	52 patients	11 screws in 5 patients total loosening 9.6%	67 patients	79 screws in 16 patients total loosening 23.9%

**Table 3:** Distribution of screw loosening by screws inserted on each spinal level. Screw loosening was observed in three different parts of the screws: the pedicular section, the corpus section around the screw hole, and the tip of the screw. A *p*-value less than 0.05 is considered to be statistically significant.

Surgical Level	Pedicular Loosening	Corpus Loosening		Tip Loosening	Total Loosening	Total Loosening (%)	Pedicular vs. Corpus *p*-Value	Total Loosening *p*-Value
T11	3	1	0	4	4.44%		0.620	0.166
T12	7	4	3	14	15.56%		0.746	0.031
L1	5	3	2	10	11.11%		0.719	0.665
L2	1	4	2	7	7.78%		0.200	0.040
L3	1	3	1	5	5.56%		0.351	<0.001
L4	2	5	1	8	8.89%		0.262	0.002
L5	4	4	1	9	10.00%		>0.999	0.006
Iliac	5	3	6	14	15.56%		0.719	0.001
S1	9	6	4	19	21.11%		0.777	0.964
Total	37	33	20	90				

Fisher’s exact test, chi-square test.

**Table 4:** Comparison of screw loosening in single and two-stage dynamic stabilization procedure with Dynesys system. A chi-square test was used. RR—relative risk, CI—confidence interval.

Surgical Stage	Number of Loosened Screws	Number of Integrated Screws	Total Number of Screws	Screw Loosening Rate	*p*-Value
Single-Stage	79	461	540	14.63%	*p* < 0.001 RR: 0.88, 95% CI: 0.84–0.91
Two-Stage	11	378	389	2.83%	
Total	90	839	929	9.69%	

**Table 5:** Comparison of clinical outcomes between the single-stage and two-stage dynamic stabilization followed by Visual Analog Scale (VAS) and Oswestry Disability Index (ODI) scores. Data are represented as mean ± standard deviation. Ordinary one-way ANOVA analysis was applied.

Clinical Parameters	Preoperative	Preoperative (2nd Stage)	6 Month	12 Month	24 Month	48 Month	*p*-Value
VAS							
Single-Stage	8.78 ± 2.73		3.6 ± 1.3	3.5 ± 1.0	3.2 ± 0.9	2.9 ± 0.6	<0.001
Two-Stage	8.65 ± 2.51	7.54 ± 2.05	3.8 ± 1.2	3.0 ± 1.1	2.8 ± 1.0	2.4 ± 0.6	<0.001
ODI							
Single-Stage	69.71 ± 10.82		28.2 ± 4.5	25.8 ± 3.7	23.1 ± 2.9	19.1 ± 2.2	<0.001
Two-Stage	71.23 ± 12.06	65.86 ± 11.01	28.7 ± 4.9	23.1 ± 3.3	21.4 ± 3.1	18.3 ± 2.4	<0.001

## Data Availability

The datasets used and/or analyzed during the current study are available from the corresponding author upon reasonable request.
